# Measurement feedback system implementation in public youth mental health treatment services: a mixed methods analysis

**DOI:** 10.1186/s43058-022-00356-5

**Published:** 2022-11-21

**Authors:** Corianna E. Sichel, Elizabeth H. Connors

**Affiliations:** 1 Division of Child and Adolescent Psychiatry, Department of Psychiatry, Columbia University, 1051 Riverside Drive, Mail Unit 78, New York, NY 10032 USA; 2 Division of Prevention and Community Research, Department of Psychiatry, Yale University School of Medicine, 389 Whitney Avenue, New Haven, CT 06511 USA

**Keywords:** Measurement-feedback system, Measurement-based care, Implementation outcomes, Determinants of practice, Clinician characteristics, Community-based mental health

## Abstract

**Background:**

Prior studies indicate the effectiveness of measurement-based care (MBC), an evidence-based practice, in improving and accelerating positive outcomes for youth receiving behavioral health services. MBC is the routine collection and use of client-reported progress measures to inform shared decision-making and collaborative treatment adjustments and is a relatively feasible and scalable clinical practice, particularly well-suited for under-resourced community mental health settings. However, uptake of MBC remains low, so information on determinants related to MBC practice patterns is needed.

**Methods:**

Quantitative and qualitative data from *N* = 80 clinicians who implemented MBC using a measurement feedback system (MFS) were merged to understand and describe determinants of practice over three study phases. Quantitative, latent class analysis identified clinician groups based on participants’ ratings of MFS acceptability, appropriateness, and feasibility and describes similarities/differences between classes in clinician-level characteristics (e.g., age; perceptions of implementation climate; reported MFS use; phase I). Qualitative analyses of clinicians’ responses to open-ended questions about their MFS use and feedback about the MFS and implementation supports were conducted separately to understand multi-level barriers and facilitators to MFS implementation (phase II). Mixing occurred during interpretation, examining clinician experiences and opinions across groups to understand the needs of different classes of clinicians, describe class differences, and inform selection of implementation strategies in future research (phase III).

**Results:**

We identified two classes of clinicians: “Higher MFS” and “Lower MFS,” and found similarities and differences in MFS use across groups. Compared to Lower MFS participants, clinicians in the Higher MFS group reported facilitators at a higher rate. Four determinants of practice were associated with the uptake of MBC and MFS in youth-serving community mental health settings for all clinicians: clarity, appropriateness, and feasibility of the MFS and its measures; clinician knowledge and skills; client preferences and behaviors; and incentives and resources (e.g., time; continuing educational support). Findings also highlighted the need for individual-level implementation strategies to target clinician needs, skills, and perceptions for future MBC and MFS implementation efforts.

**Conclusion:**

This study has implications for the adoption of evidence-based practices, such as MBC, in the context of community-based mental health services for youth.

Contributions to the literature

- Measurement-based care (MBC) is an evidence-based practice which entails the collection and discussion of client-reported progress during mental health treatment, for collaborative decision-making between mental health clinicians and clients and can be implemented using digital measurement feedback systems (MFS) to facilitate the gathering and analysis of measures.
- Although MBC using MFS is a relatively feasible and scalable practice for public mental health service settings such as schools and community-based clinics, we found that (a) some clinicians were more likely than others to implement MBC, (b) there were meaningful differences between these groups of clinicians (for example, in their perceptions of mental health agency support for evidence-based practice), and (c) across groups, clinicians identified similar barriers and facilitators as important in MBC implementation.
- These findings build upon recognized, specific gaps in the literature, with implications for practice, by identifying barriers and facilitators which should be prioritized when seeking to increase uptake of evidence-based practices such as MBC in usual care, youth-serving mental health settings.

## Background

Recent national prevalence studies indicate that almost 50 million—or one in six—children and adolescents (hereafter, youth) under age 18 meet the criteria for at least one diagnosable mental health condition in the USA [1], with longitudinal data indicating a discernable rise in mental health burden among youth in the USA and worldwide [2–4]. Despite documentation of this need, poor access to mental health services for youth persists as a major public health concern [1, 5, 6]. Among youth who do access mental health treatment, most do so in local school and community-based settings through the public mental health or education sectors [7]. These systems are drastically underfunded, resulting in scarce resources to support and sustain the consistent use of evidence-based practices (EBPs) by clinicians [8–12].^1^ As a result, there is substantial heterogeneity in the quality of services provided in these “usual care” settings due to inconsistent EBP implementation and a lack of detailed, descriptive information about existing practices and needed supports [11–13]. Public child-serving systems are in dire need of feasible, scalable strategies to elevate the quality of the usual care mental health treatment they provide.

The current study examines implementation outcomes of a relatively feasible, scalable clinical practice to elevate the quality of usual care mental health services for youth of varied ages and presenting concerns: the use of a digital measurement feedback system (MFS) to promote measurement-based care (MBC) [14–16]. MBC is the routine collection and use of client-reported progress measures to inform shared decision-making between the client and clinician, and collaborative treatment adjustments as needed [16]. MBC has three components: (1) collection of client-reported outcome measures to track progress on goals, (2) sharing progress data with the youth client and their parent or caregiver and discussing their perspectives on progress data, and (3) acting on those data, informed by youth and caregiver perspectives, and other information, to make collaborative decisions about the continued treatment approach [17]. MBC has been associated with greater and faster improvements in client outcomes among youth and adults alike and is consistent with an evidence-based approach to mental health service delivery [18–20]. Despite its applicability across client populations, treatment approaches, presenting concerns, and potential to improve youth mental health care quality, MBC is currently implemented by only a small fraction of usual care mental health clinicians [21]. Moreover, systematic evaluations of MBC implementation in child-serving settings, with a particular focus on how to support clinicians’ use of MBC with youth, are still needed [20].

### MBC implementation determinants

Determinants of MBC implementation, which comprise multi-level barriers and facilitators to this practice, include factors for clients (e.g., time for completing measures, symptoms or differing abilities that make MBC more or less challenging), clinicians (e.g., questions about if MBC is superior to clinical judgment, MBC knowledge and skill, administrative burden such as paperwork, questions about how the data will be used to evaluate performance), organizations (e.g., degree of guidance on measure selection, resources for training, staff retention or turnover, leadership support, organizational climate), and behavioral health care systems (e.g., incentives for MBC and recognition of MBC benefits) [19]. In reality, multi-level determinants and implementation outcomes are interrelated and interdependent, particularly for MBC, as the client experience, client-clinician interactions, and clinician professional experiences in their organizations are all nested within health care systems [22–26].

Studies examining implementation processes in health care systems underscore the importance of considering the influence of clinician characteristics and perspectives as well as organizational factors such as climate and leadership to understand the adoption and use of EBPs [27–29]. Current implementation science theory suggests that implementation strategies should be selected and designed to address specific barriers identified within a health care system for a particular practice [30]. Examinations of the effectiveness of tailored strategies are in progress and early findings are mixed with scholars in this area recommending additional research [31, 32]. The current study extends research focused solely on barriers and facilitators of MBC by examining how selected determinants that appear in the literature predict class membership of clinicians by their reported implementation of MBC using an MFS. This provides a foundation for selecting implementation strategies for clinicians or clinician groups based on their personal constellation of determinants and use patterns. Given the multitude of determinants documented to predict MBC use, we focused specifically on clinician years of experience and implementation climate to predict and describe class differences due to consistent emphasis on these determinants of evidence-based practice (EBP) adoption and implementation in the literature. Implementation climate has been shown to predict more positive attitudes toward EBPs and clinician use of EBP [33, 34]. Clinician years of experience has also been linked to EBP knowledge and attitudes [35, 36], although some findings are mixed [11, 37–39], suggesting the need for continued examination of this determinant in various samples and implementation study designs. We complemented our focus on clinician years of experience and implementation climate by incorporating qualitative data that reflects a wider range of multi-level determinants related to MBC and MFS implementation.

### Measurement feedback systems to support MBC implementation

Use of a measurement feedback system (MFS) is one proposed implementation strategy to support the adoption and implementation of MBC [40]. MFS are health information technologies that capture client-reported outcome data and provide real-time graphical displays or other user-centered features to support clinical decision-making [40, 41]. Some of the highest effect sizes of MBC on client outcomes (e.g., *d*=0.49 to 0.70) were found when clinicians and clients viewed measures together and could identify through graphical displays when the client is “off track” compared to what would be expected [18, 42–44].

Despite the promise of MFS, clinician adoption and uptake of these technologies are variable and fraught with numerous barriers [26, 45, 46]. Findings from organization- or system-wide implementations of MFS suggest that clinician collection of measures can be more readily facilitated than clinician review and use of these data to inform treatment decisions [46, 47]. A closer look at “users” versus “non-users” of an MFS rollout in the Veteran Affairs Canada indicated that “non-users” were more likely to report barriers such as time burden and difficulty using the MFS despite reporting they had adequate knowledge and skill to use MBC [47]. Given the mounting evidence of challenges to MFS implementation, there is a great need for continued research to uncover factors that will facilitate efforts to address these barriers. For example, a system-wide MFS rollout in Hawai’i indicated that MFS implementation was facilitated by clinicians’ willingness to overcome administrative barriers and perceptions that the measure itself is clinically useful to aide decision-making [48]. Although MFS implementation has been explored in adult-serving settings, there is scant literature on predictors of MFS implementation success in youth mental health service delivery contexts. One notable case study provides a detailed account of barriers and facilitators to MFS implementation based on semi-structured interviews with eighteen clinicians across two sites, comparing and contrasting clinician-reported determinants by site [26]. In the current study, we seek to build upon work such as this to understand variations of determinants based on their clinician MFS use.

### Implementation outcomes

Implementation outcomes are proximal indicators of implementation success based on specific actions taken to promote a new treatment, practice, or service [49]. Implementation outcomes include acceptability, adoption, appropriateness, costs, feasibility, fidelity, penetration, and sustainability [49]. By explicitly measuring implementation outcomes, one can track the extent to which the implementation process occurs as expected. Neither more distal service outcomes nor client outcomes will be influenced by introduction of a new practice unless the implementation process is successful. Implementation outcomes are affected by the implementation strategies selected and various dimensions of the service context, including barriers and facilitators to implementing a new practice in a particular setting [50, 51].

## Current study

Given the need for deeper understanding of implementation determinants associated with the use of MBC and MFS in youth mental health service delivery contexts, this study sought to understand how clinician characteristics and perceptions of implementation climate related to MBC implementation outcomes and determinants of practice. The goals of the current study were to (1) identify groups or “classes” of clinicians based on their self-reported MFS implementation outcomes; (2) determine if those groups were characteristically distinct; and (3) examine barriers and facilitators to MFS implementation by class to inform how to best support future adoption and use of MFS based on clinician characteristics. We measured acceptability, feasibility, and appropriateness as implementation outcomes because they are conceptually distinct yet related and often examined in formative studies as key predictors of implementation success [52].

We used a cross-sectional design to survey clinicians at the end of 1 year of full MFS implementation. Using latent class analysis (LCA), we identified clinician classes based on self-reported implementation outcomes. LCA is a probabilistic, person-centered method through which individuals are assigned to a specific class and is an appropriate method to use for the identification of groups of people based on their similarities (in contrast, variable-centered approaches, such as confirmatory factor analysis, focus on associations between variables [53]). In our study, class membership was estimated based on participant endorsements of implementation outcomes. After conducting the LCA, we examined how clinician characteristics (e.g., years of experience) and agency factors (i.e., implementation climate) were associated with class membership. Finally, we conducted qualitative coding on clinician comments about barriers and facilitators to implementation and used mixed methods analysis to expand our understanding of implementation determinants for clinicians overall, and by class. Study description and result reporting are consistent with Levitt et al.’s (2018) recommendations as described in the Mixed Methods Article Reporting Standards.

## Methods

### Study context

The data for this study were originally collected as part of a quality improvement project in partnership with a large, suburban school district in the Mid-Atlantic region (i.e., 85,000 students in 125 public schools) and their network of community-based mental health clinicians who provide services on school grounds. Services were primarily funded by public insurance reimbursement; 74% of the students served qualified for free or reduced price lunch at school, an imperfect but conventional indicator of socioeconomic disadvantage in the USA [54]. The project focused on the adoption and implementation of a new MFS to help clinicians implement MBC and more systematically track student psychosocial progress during mental health treatment. The MFS used in this study was a private label of ACORN (https://acorncollaboration.org/acorn-leadership) developed with funding from the state’s Accountable Care Organization at the time. The MFS included the Client Feedback Form, a 19-item, standardized measure of internalizing concerns, externalizing concerns, and working alliance, with child- and parent-reported versions. The measures demonstrated strong internal consistency (*α* = 0.87–0.90) and construct validity was established via the youth Outcome Questionnaire and Child Behavior Checklist [55]. Measures were collected via paper and pencil or electronically in the MFS during session, with an emphasis on in vivo review of responses with the youth and/or parent to discuss progress and adjust treatment as needed. Data collection and submission options (i.e., via paper and pencil or electronic) were provided to accommodate for variations in technology at school sites.

### Participants

Eighty clinicians from four community-based mental health agencies participated in the study. Of those, 52.5% (*n* = 42) were from one agency. All clinicians reported that their highest level of education was a master’s degree. A minority of participants (11.3%, *n* = 9) were supervising or lead clinicians (who also saw clients), and the majority (68.75%, *n* = 55) were trained in social work. Participant ages ranged from age 21–61, with a median age range of 31–40, and years of experience in behavioral health ranged from <1 year, to over 20 years, with a median of 3–5 years. See Table 1 for participating clinician characteristics.

**Table 1:** Clinician participant characteristics (*N* = 80)

	***n*** (%)
**Age (years)**	
21–30	35 (43.8)
31–40	29 (36.3)
41–50	11 (13.8)
51–60	3 (3.8)
61+	2 (2.5)
**Years working in behavioral health**	
< 1	4 (5)
1–2	11 (13.75)
3–5	27 (33.75)
6–10	22 (27.5)
11–15	8 (10)
16–20	4 (5)
20+	4 (5)
**Degree**	
Master’s degree	80 (100)
**Role**	
Clinician	71 (88.8)
Supervisor^a^	9 (11.3)
**Field**	
Professional Counseling	10 (12.5)
Social Work	55 (68.75)
Clinical or Counseling Psychology	10 (13.75)
Other	3 (3.75)
**Agency**	
1	10 (12.5)
2	17 (21.25)
3	42 (52.5)
4	11 (13.75)

^a^All participants in the current study were clinicians. Some (11.3%) had additional responsibilities as supervising clinicians, meaning that they would meet regularly with a small group of more junior clinicians (e.g., LCSW clinicians seeking supervised field experience to become a LCSW-C) in addition to carrying a caseload of child clients

### Implementation strategies and procedures

The current study was reviewed by the Yale University Institutional Review Board and approved as exempt from continuing review due to anonymous data collection. Implementation of MBC via the MFS occurred over the course of an academic school year. The year prior was a planning year in which the MFS was chosen by school district and mental health agency leadership and pilot tested to inform training, implementation strategies, and recommended frequency of data collection [56]. During implementation, clinicians received a one hour virtual training by the MFS developer, mental health agency leaders received a one hour virtual supervisors training by the MFS developer, and quarterly meetings were held with school district and agency leaders to discuss implementation progress and strategies for ongoing support. Each agency offered implementation strategies focused on fostering clinician peer-to-peer and supervisory support for using the MFS (e.g., reviewing client measures during supervision, pairing new clinicians with implementing clinicians for peer consultation on how to use the MFS), and regular discussion of successes and challenges during staff meetings. Implementation strategies started at the beginning of the school year with the virtual MFS trainings and continued throughout the school year. Clinicians reported perceptions of the acceptability, feasibility, and appropriateness (i.e., implementation outcomes) of the MFS at the end of the year. All data were collected electronically, with participants completing the quantitative questionnaire first, and then—during the same session—responding to qualitative questions.

### Measures

#### Quantitative

##### Demographic data

Clinicians self-reported demographic and professional characteristics including age, years working in behavioral health, field of training, highest degree, job role, and caseload size. To preserve clinician anonymity, race/ethnicity and gender were not collected.

##### MFS use

At the end of the implementation year, clinician use of the MFS was measured by asking clinicians to report the number of clients with whom they had completed at least one assessment, the number of clients with whom they had completed at least two assessments, the number of clients to whom they provided feedback based on MFS results, and the number of clients for whom they changed treatment approach, informed by MFS results. Although MBC as a practice is facilitated by the MFS as a tool [57], this implementation was focused on the use of the MFS for MBC, so clinician-reported implementation outcome measures such as MFS utilization and acceptability, appropriateness, and feasibility (below) referred to the MFS as the referent “practice” or “innovation.”

##### Implementation climate

Clinicians’ perceptions of the implementation climates of their agencies were assessed with the Implementation Climate Scale [58]. The ICS has demonstrated reliability and validity in measuring staff perceptions of the implementation climate within organizations [58, 59]. The ICS is an 18-item measure with six subscales (three items per subscale) developed to assess organizational support for the implementation of EBPs, in general. All items are scored on a 5-point Likert scale: 0 (“not at all”) to 4 (“very great extent”). The *focus on EBP* subscale is comprised of items addressing the degree to which an organization prioritizes the use of EBPs (e.g., “one of this agency’s main goals is to use EBPs effectively”). The *education support* subscale addresses the degree to which an organization provides educational resources and supports for clinicians’ use of EBPs (e.g., *“*this agency provides conferences, workshops, or seminars focusing on EBPs”). The *recognition* subscale assesses the degree to which individuals with expertise in EBPs are recognized within the organization (e.g., “agency staff who use EBPs are held in high esteem in this agency”). The *selection* subscale addresses the degree to which an agency hires staff who have expertise in and value EBPs (e.g., “this agency actively recruits staff who value EBP”). The *openness* subscale pertains to staff openness to change (e.g., “this agency selects staff who are adaptable”). In the current sample, the overall scale and the *focus, recognition, rewards, and openness* subscales had acceptable internal consistency (alphas between 0.70 and 0.92). However, internal consistency of the *educational support* and *selection* subscales was below the threshold (alphas of 0.52 and 0.62 respectively), although this may be related to the small number of items per scale [60]. Conservatively, findings associated with these two subscales should be interpreted with caution.

##### Implementation outcomes

Implementation outcomes were assessed using three measures with demonstrated reliability in assessing clinician-reported implementation outcomes: the Acceptability of Intervention Measure (AIM), the Intervention Appropriateness Measure (IAM), and the Feasibility of Intervention Measure (FIM [52]). Each scale is comprised of four items and each item is scored on a 5-point Likert scale ranging from 1 (“completely disagree”) to 5 (“completely agree). The AIM addresses the extent to which clinicians find an EBP to be satisfactory (e.g., “I like the MFS”); the IAM assesses clinicians’ perceptions about the fit of the EBP (e.g., “the MFS seems like a good match”), and the FIM probes clinician perceptions of the viability of EBPs (e.g., “the MFS seems implementable”).

#### Qualitative

Qualitative data were drawn from clinicians’ responses to the following four open-ended questions: *1) What do you need right now to support your use of the MFS? 2) What do you think new clinicians will need to support their use of the MFS next year? 3) What do you like about the MFS? 4) What do you not like about the MFS or recommend could be improved?* Finally, clinicians were given the option of providing additional comments, feedback, or recommendations about the implementation of the MFS in an open text box.

### Data analytic plan

The current study employed a partially mixed, sequential, equal status design with mixing occurring in the interpretation phase ([61]; Table 2). Using mixed methods allowed us to extend the scope of inquiry by using different methods to address different aspects of the topic at hand (i.e., for the purpose of expansion [62, 63]). Specifically, in phase I, we used quantitative methods to identify classes of participants based on self-reported implementation outcomes and compare clinician characteristics across classes. In phase II, determinants of practice were identified through qualitative data coding, and codes were guided by an established list of determinants [64]. Finally, mixing occurred in phase III, when quantitative and qualitative data were combined to understand the needs of different classes of clinicians, describing class differences, and providing information to inform the selection of specific implementation strategies for future implementation and research efforts.

**Table 2:** Mixed methods study phases

Phase	Data analysis	Purpose
I	Latent class analysis and characterization of emergent classes	Identify classes of clinician participants based on self-reported implementation outcomes (dependent variable) to determine if clinicians in different classes are characteristically different
II	Thematic coding	Code clinician qualitative feedback about the implementation process to identify determinants of practice (i.e., barriers and facilitators) based on an established list (65)
III	Mixing	Combine quantitative and qualitative data to understand the needs of different classes of clinicians, describe class differences and inform selection of specific implementation strategies in future research

#### Quantitative data analysis

Quantitative data analyses were conducted in SPSS Version 26 and MPlus 7. Using intraclass correlation coefficients (ICCs), we examined shared variance of participants within agency at the item and subscale levels for the Implementation Climate Scale and three implementation outcome measures [52, 58]. All ICCs were small-to-medium.^2^ Next, we conducted a series of LCAs with robust standard errors (a conservative approach to account for the nesting of clinicians in agencies [65, 66]) to identify classes of participants to better understand variations in implementation outcomes. Specifically, clinicians’ self-reported quantitative ratings of MFS acceptability, appropriateness, and feasibility were analyzed in a latent class framework to identify classes of clinicians. We conducted all LCAs to model subgroups of participants with different patterns of endorsement of implementation outcomes. To identify a final model, we compared entropy and fit statistics across models with two, three, and four classes and selected the two-class model. Although LCA is frequently used with much larger samples, there is support for use of LCA with samples as small as *N* = 30, when there are relatively few, distinctive classes [65]. We evaluated LCA solutions based on the theoretical interpretability of the classes, class size, Akaike’s Information Criterion (AIC), Bayesian Information Criterion (BIC), adjusted BIC (aBIC), entropy, Vuong-Lo-Mendell-Rubin Likelihood ratio test (VLMR LRT), and Lo-Mendell-Rubin Adjusted Likelihood Ratio Test (LMR LRT). AIC, BIC, and aBIC are relative fit statistics, with smaller values interpreted as indicating superior fit. Entropy measures class distinction, such that values of 0.8 and higher indicate acceptable class separation. VLMR and LMR LRT provide *p* values to assess if adding additional classes results in statistically significant fit improvement (Nylund et al., 2007). We used logistic regressions to understand specific predictors of class membership and identify similarities and differences in clinician characteristics at the level of the individual, including age, caseload, years of experience, clinician reports of implementation climate, and MFS utilization, across classes. As a conservative method, we adjusted for agency in all regression analyses by including agency as a covariate.

#### Qualitative data analysis

Consistent with a thematic coding approach, we established a coding scheme based on Flottorp and colleagues’ [64]  domains of determinants of practice. These domains include guideline factors, individual health professional factors, client factors, professional interactions, incentives and resources, capacity for organizational change, and social, political, and legal factors. Qualitative data were organized by question (rather than respondent) for analysis. Although our a priori coding scheme consisted only of determinants from Flottorp and colleagues, emergent themes were identified within each domain through a consensus process to contextualize our understanding and operationalization of these domains for the current practice and setting. Both authors served as coders and met weekly during the early coding process to review codes and establish interrater consensus. After all qualitative material was coded in Excel, we met and reviewed each individual code to ensure agreement and consensus.

After identifying determinants, clinician responses were coded as either “barriers” or “facilitators” for the use of the MFS. Both authors reviewed all qualitative data, and final codes were established through a consensus process which included iterative rounds of coding and discussion to ensure shared understanding of the data. When a clinician’s response indicated multiple determinants, the response was coded multiple times (e.g., if a response indicated guideline factors and individual health professional factors, both codes were applied). We were blind to clinician class membership during coding; class membership was an added variable after coding was completed. We both received doctoral-level training and supervision in qualitative methods, with experience conducting and publishing qualitative and mixed methods research [67–74]*.*

#### Mixing

Following the completion of all analyses, quantitative and qualitative results were mixed. Clinician class was added to the qualitative dataset at the clinician level, and we re-examined coded determinants to identify patterns within and between classes. Specifically, we examined the type of determinants as well as the relative frequency of barriers and facilitators. After mixing, we reviewed findings separately and then met to discuss and achieve consensus concerning patterns in the data. The goal of mixing quantitative and qualitative analyses was to understand possible differences and similarities in the barriers and facilitators experienced by different groups of clinicians.

## Results

### Latent class analysis: a two-class solution

**Fig. 1:**
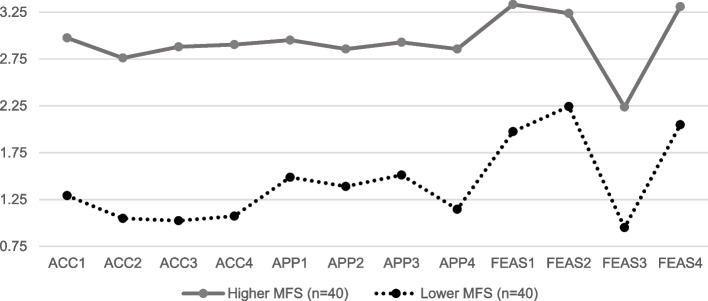
Endorsement of AIM, IAM, and FIM items for two-class solution

Entropy was acceptable for all models. Relative fit statistics (i.e., AIC, BIC, aBIC) suggested that there might be support for a more complex model; however, the non-significant *p*-values for the Vuong-Lo-Mendell Likelihood Ratio Test and the Lo-Mendell adjusted Likelihood Ratio Test indicated support for the two-class model [75]. See Table 3 for model fit statistics. Therefore, we examined patterns of endorsement of AIM, IAM, and FIM measures across classes and chose to move forward with the two-class model for parsimony (Fig. 1).

**Table 3:** Model-fit statistics comparisons

Classes	AIC	BIC	aBIC	Vuong-Lo-Mendell LR test *p*-value	Lo-Mendell adjusted LR test *p*-value	Entropy	*N* per class
2	2346.34	2572.63	2273.06	0.26	0.26	0.98	I = 40 II = 40
3	2182.97	2523.60	2072.67	0.58	0.59	0.99	I = 32 II = 19 III = 29
4	2055.08	2510.04	1907.75	0.39	0.39	0.99	I = 6 II = 30 III = 25 IV = 19

AIC, BIC, and aBIC are relative fit statistics, with smaller values indicating better comparative fit. Entropy measures class separation with entropy ≥ 0.80 indicating acceptable class distinction. The Vuong-Lo-Mendell LR test and the Lo-Mendell adjusted LR test provide *p* values for use in assessing if adding additional classes will result in a statistically significant improvement in fit [75]

In the two-class model, there were 40 clinicians in each class. The first class, the “Higher MFS Self-Reported Implementation Outcomes group,” (referred to as “Higher MFS” going forward) viewed the MFS more positively and was comprised of clinicians who gave it higher ratings of acceptability, ranging from 2 to 4 on the AIM, *M =* 2.89, *SD =* 0.59; appropriateness, ranging from 1.5 to 4 on the IAM, *M =* 2.91, *SD =* 0.57; and feasibility ranging from 2.50 to 3.75, *M=*3.05, *SD=*0.44, than clinicians in the second class, the “Lower Self-Reported MFS Implementation Outcomes” (referred to as “Lower MFS” going forward; Fig. 1). Among clinicians in the Lower MFS class, ratings of acceptability on the AIM ranged from 0 to 3, *M =* 1.11, *SD =* 0.84; ratings of appropriateness on the IAM ranged from 0 to 3, *M =* 1.38, *SD =* 0.85; and ratings of feasibility on the FIM ranged from 0.5 to 3.75, *M = 1.78*, *SD =* 0.73. In general, the two classes followed similar patterns of responses on the AIM, IAM, and FIM, with Higher MFS clinicians agreeing more than Lower MFS clinicians. We inspected the distribution of classes across agencies using chi-square tests and found no significant differences, with two exceptions: clinicians in agency 2 were significantly less likely to be in class I compared to clinicians in agency 3 *χ*^2^(1, *n* = 59)= 7.13, *p =* 0.01, and clinicians in agency 4 *χ*^2^(1, *n* = 28)= 4.50, *p =* 0.03. Adjusting for agency as a covariate, there were no differences across classes in age, years in behavioral health, or caseload (Table 4).

**Table 4:** Class comparisons on clinician characteristics, climate perceptions, and MFS use

	Total sample	Higher MFS	Lower MFS		
	M (SD)	M (SD	M (SD	*aOR [95% CI]*	*p*
> 30 years old	44%	47%	40%	1.76 [0.68, 4.59]	0.25
> 5 years of BH experience	48%	45%	50%	0.92 [0.37, 2.32]	0.87
Total caseload	20.53 (10.22)	20.37 (11.35)	20.70 (9.09)	1.00 [0.96, 1.05]	0.89
Implementation Climate Scale	2.22 (0.77)	2.48 (0.65)	1.95 (0.8)	2.80 [1.37, 5.72]	0.01
Implementation Climate Subscales					
Focus on EBP	2.77 (0.87)	3.03 (0.69)	2.51 (0.96)	2.36 [1.26, 4.43]	0.01
Educational Support for EBP	2.02 (0.86)	2.28 (0.76)	1.74 (0.89)	2.36 [1.26, 4.42]	0.01
Recognition for EBP	1.92 (1.04)	2.31 (0.90)	1.50 (1.02)	2.40 [1.37, 4.19]	<0.01
Rewards for EBP	2.55 (0.87)	2.81 (0.72)	2.29 (0.94)	2.18 [1.18, 4.02]	0.01
Selection for EBP	2.76 (0.77)	2.98 (0.66)	2.54 (0.83)	2.27 [1.13, 4.55]	0.01
EBP Openness	0.79 (1.02)	0.88 (1.02)	0.70 (1.03)	1.17 [0.72, 1.19]	0.53
MFS Use^a^					
Average rate of cases completed at least 1 assessment	0.85 (0.35)	0.89 (0.17)	0.82 (0.46)	1.53 [0.38, 6.14]	0.55
Average rate of cases completed at least 2 assessments	0.71 (0.42)	0.76 (0.26)	0.65 (0.51)	1.68 [0.53, 5.36]	0.38
Average rate of cases provided feedback based on MFS	0.40 (0.40)	0.59 (0.39)	0.23 (0.32)	10.32 [2.61. 40.87]	<0.01
Average rate of cases changed treatment approach based on MFS	0.28 (0.59)	0.40 (0.67)	0.17 (0.49)	2.03 [0.65, 6.30]	0.22

All comparisons were made with logistic regressions, adjusting for agency

^a^Average rates of MFS use were calculated by dividing number of cases reported by each clinician by their total caseload (e.g., if a clinician reported completing at least one assessment with two cases and they had a caseload of 10 their *rate of cases completed at least 1 assessment* would be 0.20)

#### Perceptions of implementation climate

Clinician’s implementation climate ratings of their agencies were significantly different across classes (Table 4), such that Higher MFS participants rated their agency’s climate as more supportive of EBPs (*M* = 2.48, *SD* = 0.65) than Lower MFS participants (*M* = 1.95, *SD* = 0.80, Adjusted Odds Ratio (*aOR*) = 2.80 [1.37, 5.72], *p* = 0.01). There were also significant differences on five of the six the ICS subscales. Specifically, Higher MFS participants reported significantly more organizational focus on EBP (*M* = 3.03, *SD* = 0.69) than Lower MFS participants (*M* = 2.51, *SD* = 0.96; *aOR* = 2.36 [1.26, 4.43], *p* = 0.01), and significantly more educational support for EBP (*M* = 2.23, *SD* = 0.76) compared to Lower MFS participants (*M* = 1.74, *SD* = 0.89; *aOR* = 2.36 [1.26, 4.42], *p* = 0.01). Clinicians in the Higher MFS group reported significantly higher levels of organizational recognition for EBP (*M* = 2.31, *SD* = 0.90) compared to Lower MFS participants (*M* = 1.50, *SD* = 1.02; *aOR* = 2.40 [1.37, 4.19], *p* < 0.01) and significantly more rewards for EBP (*M* = 2.81, *SD* = 72) than Lower MFS participants (*M* = 2.29, *SD* = 0.94; *aOR* = 2.18 [1.18, 4.02], *p* = 0.01). Higher MFS clinicians also had significantly higher ratings on the selection for EBP subscale, indicating that Higher MFS participants were more likely to perceive their agencies as selecting staff with strong backgrounds in EBP (*M* = 2.98, *SD* = 0.66), compared to Lower MFS participants (*M* = 2.54, *SD* = 0.83; *aOR* = 2.27 [1.13, 4.55], *p* = 0.01). Subscales addressing educational support for EBP and selection for EBP had low internal consistency, and findings should therefore be interpreted with caution. There were no significant differences between classes in clinician’s endorsement the Openness subscale, indicating no difference across classes in clinician perception of agency likelihood to select staff who were open to EBP.

#### MFS use

Clinicians reported one difference in MFS use between classes: adjusting for agency, Higher MFS participants had significantly higher rates of providing feedback based on MFS data (*M* = 0.59, *SD* =0.49) compared to Lower MFS participants (*M* = 0.23, *SD* = 0.32; *aOR* = 10.32, *p <* 0.01; Table 4). Adjusting for agency, there were no significant differences between classes on the rate completion of at least 1 or at least 2 assessments, or clinician rates of changing treatment approach based on MFS results.

### Qualitative results

Four domains of determinants emerged most frequently in clinician’s responses to open-ended questions about MFS implementation: guideline factors, individual professional factors, client factors, and incentives and resources (see Table 5). Thus, we focused our qualitative analyses on these areas. Whereas quantitative results focus on factors (i.e., possible determinants) at the clinician level to distinguish classes (particularly as implementation climate varied more at the clinician than agency level, in this sample), our qualitative results reflect clinician perspectives of determinants at multiple levels, which are indicated in the subheadings below.

**Table 5:** Percentages and counts of implementation determinants identified in qualitative data

Determinant	Total sample	Higher MFS	Lower MFS
	*n* (%)	*n* (%)	*n* (%)
**Number of determinants**	422	222	200
**Determinants identified as facilitators**	192 (45.50)	120 (54.05)	72 (36.00)
**Guideline factors** *Recommendation to use MBC via the MFS (e.g., clarity, appropriateness, feasibility)*	194 (45.97)	96 (43.24)	98 (49.00)
**Individual professional factors** *Clinician characteristics (e.g., knowledge, skills, self-efficacy, cognitions/attitudes/emotions about MBC and the MFS)*	86 (20.38)	49 (22.07)	37 (18.50)
**Client factors** *Client characteristics (e.g., knowledge, beliefs, perceptions, preferences, behavior, needs)*	77 (18.25)	47 (21.17)	30 (15.00)
**Incentives and resources** *Availability of resources necessary for clinicians to implement, incentives and disincentives, assistance for clinicians, continuing education, quality assurance*	53 (12.56)	24 (10.81)	29 (14.50)
**Other** *Examples: factors associated with organizational capacity for change; professional interactions; social, political, legal factors*	12 (2.84)	6 (2.70)	6 (3.00)

A total of 422 unique mentions of determinants were found in the qualitative data; of those, five were classified as neither barriers nor facilitators

#### Guideline factors (EBP level)

Guideline factors are various aspects of how clinicians were recommended to use MBC via the MFS, which we operationally defined as the clarity, strength, appropriateness, and feasibility of the Outcome Rating Scale in the MFS *as well as* the MFS itself [46]. Therefore, qualitative results in this section reflect clinicians’ feedback about both the progress measure and the feedback system. All agencies had the same expectations for their school-based clinicians, which was to collect and discuss the Outcome Rating Scale as often as possible, up to every session, with all students and families served. Forty-six percent of determinants identified in the data were guideline factors. Clinicians identified various guideline factors as influential in their use of the MFS, including compatibility with existing clinical practices, effort required to change or adhere to the recommendation, feasibility, clarity, and the evidence supporting the recommendation. For example, clinicians expressed a desire for problem area-specific measures for progress monitoring instead of a global outcome measure, and the preference to choose unique measures for each client based on clinical judgment. One clinician wrote: “I would rather be using pre/post testing scales or client rating scales that focused on the specific issues our clients are facing such as [a] specific depression inventory, trauma rating scales, etc.” Frequency of recommended assessment arose as an important consideration in the guideline domain, with some clinicians sharing that they would prefer to administer the measure with less regularity. As one clinician explained, “I would be much more comfortable with using it once every 2-3 months… Monthly administration means that we spend about 20% of our time every month completing this.” Other aspects of the guideline that clinicians described liking included the ease of navigating the tool, the enhanced ability to monitor progress over time, the opportunity to get feedback from clients, and how the MFS enabled them to focus on evidence-based outcomes.

#### Individual professional factors (clinician level)

Individual professional factors are clinician characteristics such as knowledge, skills, self-efficacy, and attitudes about MBC and the MFS [46]. These accounted for 20% of identified determinants, with knowledge and skills, attitudes, and professional behavior [65] emerging as particularly important. For example, clinicians emphasized familiarity with, and knowledge of the MFS as key for implementation, and multiple clinicians reported that the initial MFS training they received was sufficient to equip them with the necessary understanding. Professional behavior also emerged as influential. Clinicians emphasized the importance of being organized, creating systems for themselves so that they would remember to implement the MFS, and carving out time to review findings first by themselves, and then with clients, to guide treatment. Clinician attitudes and perceptions were also highlighted as multiple clinicians described a desire for more autonomy, and disliking guidelines that required them to implement certain protocols for all clients. Relatedly, some clinicians expressed doubts about their clients’ ability to answer reliably. For example, one clinician noted, “I have a couple of girls who are very extreme in their perception of things. This makes it difficult because they will answer with the highest number, even if that is not necessarily the case.”

#### Client factors (client level)

Client needs, knowledge and beliefs, preferences, and motivations about the MFS and its outcome measure accounted for 18% of identified determinants. Multiple clinicians working with younger clients reported that the lack of adjusted, age-appropriate measures posed a significant challenge to implementation of the MFS. Additionally, some clinicians reported that their clients did not like the MFS. For example, a clinician noted: “I have tried to make [the MFS] fun for my kids by turning it into a game with a ball, but they still get annoyed that they have to do it.” Others reported that the MFS allowed clients to report problems that might not otherwise have arisen during treatment. For example, one clinician noted that the MFS helped to improve rapport and engagement, and another wrote, “it's helpful to quickly assess where clients are with their symptoms. At times clients will not identify any stressors or challenges, but this will be identified in the survey [and]… would otherwise be missed.” Similarly, a different clinician noted that the MFS helped to inform the direction of treatment, to better meet the needs of youth: “I have had many sessions change because of a discussion based on an answer they had to on [the MFS], and I don't think I would have found that information if we had just done a normal session.”

#### Incentives and resources (agency level)

Incentives and resources included those necessary for clinicians to use the MFS, as well as incentives, disincentives, and supports for implementation (e.g., assistance for clinicians, continuing educational support; 47). Thirteen percent of the identified determinants were incentives and resources. Specifically, clinicians identified the availability of necessary resources, assistance for clinicians, and continuing education to be particularly important. Time was the most frequently identified resource that clinicians mentioned. For example, one clinician reported, “I have so [many] other things to do for my job that I'm unable to use this tool to my or the client’s advantage. Unfortunately, it’s more of a requirement to meet… I haven’t had the time to go into the system [to] review the data for trends, on any of my 21 cases.” Clinicians also described a desire for someone else to help with data entry/management, and scheduling systems that would automatically create reminders for monthly implementation. Finally, some clinicians expressed a desire for continued educational support, such as refresher trainings, and examples of how to more seamlessly incorporate MBC and the MFS into treatment sessions.

### Mixing

By mixing our quantitative and qualitative data, we found that 55% of the determinants identified by Higher MFS participants were framed as facilitators that helped them implement MBC and use the MFS. In contrast, 37% of the determinants identified by Lower MFS participants were framed as facilitators. Specifically, when we coded qualitative data blind to clinician class, we identified 120 instances of facilitators and 100 instances of barriers in data from Higher MFS participants, compared to 72 instances of facilitators and 125 instances of barriers in data from Lower MFS participants. Table 5 provides an overview of the types of determinants identified by class, and percentages of instances in which each determinant was identified based on the total determinants identified.

The most common four determinants of practice identified in the qualitative data were identified across both classes. Although the Higher MFS participants reported more determinants than the Lower MFS participants, clinicians across both classes endorsed the same types of determinants. However, the fact that, descriptively, a greater proportion of the determinants identified by participants in the Higher MFS group were facilitators, compared to those identified by Lower MFS participants, suggests additional support for the two-class model: across both the qualitative and quantitative data clinicians in the Higher MFS group reported more support for MBC and the MFS.

## Discussion

In the context of an MFS implementation project within publicly funded, community-based youth mental health treatment services, we found support for two classes of clinicians. We tentatively refer to them as “Higher MFS” and “Lower MFS” participants, based on differences in clinician-reported implementation outcomes (i.e., perceptions of appropriateness, acceptability, and feasibility of the MFS and its outcome measure) and patterns of MFS use. Identifying these classes proved useful in several ways to deepen our understanding of different types of implementation experiences and perceptions that clinicians reported at the end of a full year of implementation. First, adjusting for agency, Higher MFS participants tended to view their organizational settings as more supportive of EBP in general, including higher levels of agreement that their agencies focused on EBP, recognized and rewarded clinicians for using EBP, provided educational support for EBP, and selected clinicians with experience, skills, and training in EBP (of note, the two latter subscales achieved below threshold reliability, and findings may therefore be interpreted with caution). Both groups reported similar data collection rates using the MFS, and higher rates of measure collection compared to rates of providing feedback or using MFS data to inform changes to treatment course. Previous MBC studies show similar trends: using data and adjusting treatment based on data is often the most difficult practice to change in implementation [47, 76–78]. Yet, even adjusting for agency, Higher MFS participants reported significantly higher rates of providing feedback based on the MFS, compared to the Lower MFS participants.

Findings from our qualitative analyses provided additional support for the two classes, indicating that participants in the Higher MFS group reported a higher proportion of facilitators compared to Lower MFS participants. However, types of determinants most commonly noted were the same across classes. Of note, this is not to say that implementation differences between the classes are solely due to intrapersonal clinician factors; indeed, clinicians in both classes identified similar multi-level determinants influencing their practices and those in the Higher MFS group experienced more facilitators and fewer barriers. Prior research also indicates that clinician and agency-level determinants likely interact in reality to influence implementation outcomes [79]. Our goal with this study was to center the clinicians’ perspective to understand their experiences and multi-level determinants, but future work might look to mix different stakeholder perspectives to triangulate that of clients, supervisors, leaders, and clinicians. Interestingly, qualitative results of determinants did not surface elements of the school context as much as we anticipated. This may be because the clinicians in this sample are community-partnered, which means their organizational context for implementation is a blend of their agency as the employer and school site(s) as the practice setting, so they may not face as many school-context-specific barriers to implementation as their school-employed counterparts [68, 71]. Contextual factors influencing implementation of measurement-based care and other evidence-based practices in schools, by various types of school-based clinicians, is an important area of future inquiry.

Our results indicate that clinician perceptions of implementation climate are clearly linked to clinician-reported implementation outcomes. Prior studies [80, 81] indicate the importance of clinician attitudes toward standardized assessments. Although such attitudes were not explicitly measured in the present study, our findings reflect the complex interplay between clinician perceptions and behaviors. Clinicians in the Higher and Lower MFS classes were distinguished not only by their rates of providing feedback and perspectives about the MFS, but also by their viewpoints on implementation climate. As demonstrated by small-to-medium ICCs, clinician’s perspectives about implementation climate varied in part by clinician. This suggests, perhaps, that implementation strategies targeting individual clinician perspectives and behaviors, rather than, or in addition to, agency climate, may be important in influencing the uptake of MBC and use of MFS in the future. In fact, there is a history of strategy tailoring at the organization, site, or agency level [32, 82], but our results indicate that strategy tailoring may need to also occur at the clinician level. Prior work from our team underscores the significant amount of implementation variation at the individual clinician level that is not well accounted for by our traditional measures of clinician knowledge, perceptions, and professional characteristics [77]. Tailoring to the clinician level is admittedly a more costly approach to tailoring than at the organizational level [83], so perhaps a phased approach to tailoring whereby strategies are first selected for the organization and then further customized at the clinician level for lower MFS implementers could prove to be more resource-effective.

### Implications for MBC in youth service settings

In all settings, including those that serve youth, implementation outcomes are influenced by multilevel and interrelated determinants regarding the recommended practice, setting factors, and characteristics of the individuals implementing. Systematic methods to identify the determinants that matter most can inform which implementation supports or strategies are provided. Coding implementation data by determinant using a pre-existing checklist, as we did in this study, can supply actionable, real-time information to help agency leadership, trainers, coaches, and project directors to select and provide appropriate, tailored implementation strategies. In fact, the CFIR-ERIC Implementation Strategy Matching Tool [51] is practical, free tool, accessible online, that identifies implementation strategies matched to determinants. Indeed, the community-based settings in which youth most frequently receive mental health care often have very limited resources, further underscoring the importance of identifying the most important and influential strategies to support implementation.

Guideline factors emerged as particularly critical in our study, indicating the importance of the recommendation or practice itself. This suggests that targeting intervention characteristics, such as those that promote clarity and adaptability of a measure and/or the MFS, identifying and preparing champions to support implementation in their clinical settings, and capturing and sharing local knowledge about the measure and MFS, are strategies likely to increase implementation of these practices in youth-serving, community-based mental health agencies [51]. Similarly, strategies addressing clinicians’ knowledge and beliefs about the intervention, self-efficacy in their capability achieve implementation goals, and commitment to implementing the MFS in a sustained manner [51] will be important to promote the uptake and sustainment of MBC and use of the MFS.

### Limitations and future directions

Several limitations should be noted. First, clinicians in agency 2 were significantly less likely to be in the Higher MFS class compared to those in agencies 3 and 4. This may indicate that unmeasured agency-level factors were important, perhaps in some agencies more so than others. Given this finding, we used multiple quantitative methods to mitigate the clustering effects of agency on clinician outcomes (i.e., LCA with robust standard errors and adjusting for agency in quantitative between-class analyses). A second limitation is that qualitative data on barriers and facilitators may have been influenced by the wording of the qualitative questions. Indeed, barriers and facilitators, as identified in this study, are essentially “mirror images” of each determinant domain. For example, clinicians identified lack of time as a barrier; ample time would therefore be a facilitator. Future research may consider deemphasizing the distinction between barriers and facilitators, for example by focusing on facilitators. Third, our classes were based on clinician-reported acceptability, feasibility, and appropriateness as leading outcomes for this type of work and may reflect high and low response pattern tendencies. However, the fact that there were other systematic differences between the classes provides support for the existence of real underlying between-group differences. Fourth, future research addressing MBC and MFS implementation efforts could examine other, more distal implementation outcomes such as fidelity (which may in fact be predicted or preceded by acceptability, implementation, and appropriateness [84]) as well as elicit feedback not only from clinicians but also clients and agency leadership. In our study, all client factors were clinician reported, and given evidence of the potential importance of agency-level effects, it will be important to understand the perspectives of agency leaders in future work addressing MBC and MFS implementation efforts. Relatedly, agency factors were not comprehensively assessed qualitatively. Fifth, although there are many strengths associated with our person-centered quantitative methodology, it is possible that by grouping clinicians into classes we failed to detect granular differences in associations between implementation outcomes and other study variables. Finally, our study was cross-sectional, relying on clinician’s retrospective self-report of MFS use, and did not address causation, incorporate contemporaneous reports of MFS utilization (which would likely be more accurate), or address possible trajectories in change of clinician reports of implementation outcomes over time, which are areas for future possible research.

## Conclusions

By investigating clinician factors associated with the implementation of MBC via a MFS, this study has important implications for the adaptation of EBPs in the context of community-based, “usual care” mental health services for youth. For example, our findings suggest that there are meaningful differences between clinicians, such that some are more likely to rate an MFS as acceptable, appropriate, and feasible. Notably, although we identified classes of clinician perceptions of implementation climate, these varied significantly at the clinician level, controlling for agency. This underscores the potential need to target interventions at the individual clinician level, and we are hopeful that this work and continued efforts to understand variation in clinician experiences with MBC implementation and perceived implementation climate can inform targeted, future implementation supports and solutions.

## Data Availability

The datasets analyzed during the current study are not publicly available as data were gathered as part of a quality improvement project but are available from the second author on reasonable request.

## Abbreviations

MBC: Measurement-based care
MFS: Measurement feedback system
EBP: Evidence-based practice
LCA: Latent class analysis
ICS: Implementation Climate Scale
AIM: Acceptability of Intervention Measure
IAM: Intervention Appropriateness Measure
FIM: Feasibility of Intervention Measure
ICC: Interclass correlation coefficient

## Footnotes

1 See Hoagwood and colleagues’ (2001) review for a history of evidence-based practices in child and adolescent mental health services in the United States, including treatment interventions and settings (10).

2 Item level ICCs ranged from 0.002 to 0.106 and subscale level ICCs ranged from 0.019 to 0.080.
